# Differential Microbial Composition of Monovarietal and Blended Extra Virgin Olive Oils Determines Oil Quality during Storage

**DOI:** 10.3390/microorganisms8030402

**Published:** 2020-03-13

**Authors:** Biagi Angelo Zullo, Gino Ciafardini

**Affiliations:** Department of Agricultural, Environmental and Food Sciences, University of Molise, Via De Sanctis, I-86100 Campobasso, Italy; ciafardi@unimol.it

**Keywords:** extra virgin olive oil, microbiota, oil blending, oil-born yeasts, olive oil quality

## Abstract

Extra virgin olive oil (EVOO) contains a biotic fraction, which is characterized by various microorganisms, including yeasts. The colonization of microorganisms in the freshly produced EVOO is determined by the physicochemical characteristics of the product. The production of blended EVOO with balanced taste, which is obtained by blending several monovarietal EVOOs, modifies the original microbiota of each oil due to the differential physico-chemical characteristics of the blended oil. This study aimed to evaluate the effect of microbial composition on the stability of the quality indices of the monovarietal and blended EVOOs derived from Leccino, Peranzana, Coratina, and Ravece olive varieties after six months of storage. The yeasts survived only in the monovarietal EVOOs during six months of storage. *Barnettozyma californica*, *Candida adriatica*, *Candida diddensiae*, and *Yamadazyma terventina* were the predominant yeast species, whose abundance varied in the four monovarietal EVOOs. However, the number of yeasts markedly decreased during the first three months of storage in all blended EVOOs. Thus, all blended EVOOs were more stable than the monovarietal EVOOs as the abundance and activity of microorganisms were limited during storage.

## 1. Introduction

Extra virgin olive oil (EVOO) is produced by directly subjecting the olive fruit to mechanical extraction without any further refining process. Globally, EVOO is one of the oldest vegetable oils known for its sensory and nutritional value [1]. According to the European Food Safety Authority (EFSA), the phenols in virgin olive oil protect the blood lipids from oxidative stress [2]. The health benefits of EVOO are attributed to its abiotic fraction, which is characterized by phytochemicals, such as tocopherols, carotenoids, and phenolic compounds [3,4,5]. Previous microbiological studies have demonstrated that freshly produced olive oil contains a biotic fraction, which is characterized by several microorganisms, including yeasts [6]. The yeasts in the EVOO are mainly derived from the carposphere of the olives [7]. Additionally, the yeasts can be derived from the mill plant during the extraction process [8]. Some yeast species in freshly produced EVOO do not have a long lifespan, whereas other species survive and become the predominant microbiota in the olive oil. Several yeast species in the freshly produced EVOO can remain active during the storage period and can improve or deteriorate olive oil quality, depending on their metabolic activity [9]. Recent studies have demonstrated that the presence of some yeast species, such as *Candida adriatica*, *Nakazawaea wickerhamii*, and *Candida diddensiae* may deteriorate the sensory attributes of olive oil during storage. However, the sensory attributes of EVOO containing specific *C. diddensiae* yeast strains do not deteriorate even after four months of storage [10]. Yeast population density, strain, and enzymatic activity are reported to determine EVOO chemical composition [11,12]. However, the chemical composition of EVOO can influence the survival of some yeast species during storage [13]. The high concentration of polar phenolic compounds in EVOO negatively affects the survival of some yeast species, such as *Candida parapsilosis* [14]. The fatty acid and triglyceride contents in EVOO can also inhibit the growth of several yeast strains. Several yeast species, such as *Meyerozyma guilliermondii*, *C. parapsilosis*, and *C. diddensiae* are reported to exhibit concentration-dependent sensitivity to linoleic acid [15]. Oil producers generally produce the following three types of olive oils: monovarietal EVOO, blended EVOO, and olive oil mixed with other vegetable oils, such as sunflower seed oil and grape seed oil. The flavor of monovarietal EVOOs is determined by the genetic characteristics of the olive tree and the pedo-climatic factors of the production area [16]. These oils meet the needs of the niche market in countries like Italy, where more than 700 varieties of olives are found. However, the blended EVOOs are produced by trained blenders by combining the aromatic profile of various oils. Additionally, the blended EVOOs are produced in sufficient quantities with a balanced taste to meet the demands of the international market. Most super-market brands of EVOO are blended with oils from many different cultivars, regions, and even countries. The comparative microbiological analysis of EVOOs extracted from a single olive variety and EVOOs extracted from multiple olive varieties revealed the prevalence of single yeast species only in the monovarietal EVOOs [17]. However, some oil producers blend the EVOOs extracted from different varieties of olive fruits to obtain a consistent taste profile. The effect of yeast species from each monovarietal EVOO on quality of the blended oil is not well understood. Changing the physicochemical characteristics of the monovarietal EVOOs during blending can affect the composition of the oil microbiota. Conversely, the microbial metabolic processes during storage may differential affect the quality of blended and monovarietal EVOO. This study aimed to analyze the abundance of yeasts in monovarietal and blended EVOOs and to evaluate their effect on oil quality during storage.

## 2. Materials and Methods

### 2.1. Production of Monovarietal EVOO for Blending

Monovarietal EVOOs were extracted from the ancestral Leccino, Coratina, Peranzana, and Ravece olive varieties, which have a several-hundred-years history of usage in Central-Southern Italy. During the experimental season, the olives were not subjected to insecticide treatment. The *Bactrocera oleae* (Rossi) infection rate among the fruits harvested from all the varieties was in the range of 10–20%. The homogeneous masses of approximately 300 kg of healthy olives from the same rural area were separately processed within 12 h of harvesting. The leaves and other materials were removed and the olives washed in fresh tap water. The fruits were crushed in a grinder at 2000 rpm. The paste was subjected to malaxation for 20 min at 27 °C. Next, the paste was moistened using a small amount of tap water. The oil was separated from other fruit components by double extraction through horizontal (decanter) and vertical centrifugation. The fresh EVOOs (50 L) extracted from each variety were stored separately in four batches. The EVOOs were immediately subjected to physical (suspended solid and water contents) and microbiological analyses. The four batches of monovarietal oils were allowed to settle for 30 days in a dark place at 12–13 °C before blending.

### 2.2. Physicochemical Analysis

Physicochemical analysis was performed to determine the suspended solid and water contents of the freshly extracted olive oil and the phenolic profile of the monovarietal EVOOs. The suspended solid content was assessed using 50 g of olive oil sample. The sample was filtered under reduced pressure through a 0.45 µm pre-weighed and oil-wetted nitrocellulose filter (Ministart NML-Sartorius, Göttingen, Germany). Each analysis was repeated four times. The water content of the olive oil samples was assessed following a protocol described by Ciafardini and Zullo [9]. The water content of the olive oil samples was determined using the 37858 HYDRANAL-Moisture Test Kit (Sigma-Aldrich, Seelze, Germany), following the manufacturer’s instructions. The phenolic compounds in the monovarietal EVOOs extracted from Leccino, Peranzana, Coratina, and Ravece olive varieties were evaluated by high-performance liquid chromatography (HPLC) analysis. The HPLC analysis was performed in an Agilent 1200 liquid chromatographic system equipped with a diode array UV detector and C18 column (4.6 mm i.d. × 250 mm; particle size 5 μm) (Phenomenex, Torrance, CA, USA) coupled to a C18 guard column (4 × 3.0 mm; Phenomenex). The mobile phases used in the HPLC analysis were water/acetic acid (97:3, *v v^−^^1^*) (solvent A) and methanol/acetonitrile (50:50, *v v^−^^1^*) (solvent B). The elution was performed at a flow rate of 1.0 mL min^−^^1^. The solvent gradient was changed as follows: from 95% (A) and 5% (B) to 70% (A) and 30% (B) in 25 min; 65% (A) and 35% (B) in 10 min; 60% (A) and 40% (B) in 5 min; 30% (A) and 70% (B) in 10 min; and 100% (B) in 5 min, followed by 5 min of maintenance. The chromatograms were acquired at wavelengths of 240 and 280 nm. The compounds were identified and quantified based on the retention time and absorption at different wavelengths. The analysis was repeated three times for each olive oil sample.

### 2.3. Laboratory Blending

The blending of oils was performed in a laboratory using the EVOOs extracted from the Leccino, Peranzana, Coratina, and Ravece varieties after the EVOOs were subjected to sedimentation for 30 days. In total, 24 hermetically sealed commercial canisters containing 5 L of EVOO were used. The blending was performed as follows: Leccino EVOO blended with Peranzana EVOO (LP, ratio 1:1); Coratina EVOO blended with Ravece EVOO (CR, ratio 1:1); Leccino EVOO blended with Peranzana and Ravece EVOOs (LPR, ratio 1:1:1); Leccino EVOO blended with Peranzana, Coratina, and Ravece EVOOs (LPCR, ratio 1:1:1:1). The monovarietal EVOOs extracted from Leccino, Peranzana, Coratina, and Ravece varieties were included as controls. Each blending was repeated three times. The EVOO samples were collected from each 5 L canister at the beginning of the experimentation (zero time) and after 3 and 6 months of storage at 12 °C.

### 2.4. Microbiological Analysis of EVOOs during Storage

Microbiological analysis was performed using EVOOs extracted from the Leccino, Peranzana, Coratina, and Ravece olive varieties immediately after extraction. The blended EVOO samples were analyzed at the beginning of experimentation (zero time), and after 3 and 6 months of storage at 12 °C in a dark place. Briefly, 20 mL of oil samples were micro-filtered through a 0.45 µm sterile nitrocellulose filter. The nitrocellulose filter used to capture each sample was then transferred into a 25-mL sterile beaker and homogenized using a Turrax model T25 homogenizer (IKA, Milan, Italy) in a sterile physiological solution. Finally, the initial weight of each sample was reconstituted through the addition of a sterile physiological 0.9% (*w v^−1^*) NaCl solution. The solution was then subjected to 10-fold serial dilution. The bacteria were analyzed in the plate count agar standard (PCAS) medium (Oxoid, Basingstoke, Hampshire, England). The samples (0.2 mL of the 10-fold serially diluted solution) were plated in the PCAS medium and incubated aerobically for 3 days at 28 °C. The molds were evaluated in the oxytetracycline glucose yeast extract agar medium (Oxoid) supplemented with 100 µg mL^−1^ gentamicin and 100 µg mL^−1^ chloramphenicol. The molds were counted after 7 days of incubation at 28 °C. The yeasts were analyzed in the MYGP agar medium, whose composition is as follows: 3 g yeast extract (Biolife, Milan, Italy), 3 g malt extract (BBL, Cockeysville, MD, USA), 5 g phytone powder (BBL), 10 g D-glucose (Merck, Darmstadt, Germany), and 1000 mL distilled water, pH 7 [18]. The MYGP agar medium was supplemented with tetracycline (20 mg L^−1^) to inhibit bacterial growth. The serially diluted sample (0.2 mL) was spread-plated onto the MYGP agar medium for colony counting in triplicate. The yeast colonies were counted after 5 days of incubation at 30 °C and recorded as the colony forming unit (CFU). The colonies were then transferred into several MYGP agar plates (master plates) and used for further analysis.

### 2.5. Dynamics of EVOO Yeast Species during Storage

The yeast strains isolated from the EVOO samples were identified by screening a high number of colonies grown on a specific chromogenic medium. Based on the physiological properties of the isolated yeasts, colored compounds are formed around the yeast colonies. All yeast colonies isolated from the master plates were inoculated into the CHROMagar Candida medium (BBL, cod. 4354093, Heidelberg, Germany). The colony morphology of approximately 3000 colored yeast colonies was assessed after 7 days of incubation at 30 °C [19]. All yeast colonies inoculated in the chromogenic medium were divided into five homogeneous chromogenic groups as follows: red bordeaux center with a white exterior; uniform red; fire red center with a white exterior; uniform brown; uniform white; and uniform bluish. From each chromogenic yeast colony group, 20 isolates were randomly chosen and used for subsequent identification tests.

### 2.6. Identification of Yeast Species

The selected yeast colonies that belong to different chromogenic groups were subjected to genetic analysis. The yeast strains were identified at the species level by sequencing the D1/D2 region (approximately 600 bp) of the large (26S) ribosomal subunit gene using the NL1 and NL4 primers, following the protocols described by Kurtzman and Robnett [20]. The ribosomal gene sequence of yeast strains amplified by the NL1 primer was compared with published yeast sequences available in the public sequence databases (GenBank + EMBL+DDBJ+PDB) using a BLAST search on the National Center for Biotechnology Information (NCBI) website (http://www.ncbi.nlm.nich.gov/blast).

### 2.7. Enzymatic Activity in the Predominant Yeasts in The EVOO

Enzymatic tests were performed using 20 yeast strains, which belong to the following four different species isolated from the EVOOs and identified by sequencing the D1/D2 region of the ribosomal subunit (26S) gene: *Barnettozyma californica*, *Candida adriatica*, *Candida diddensiae*, and *Yamadazyma terventina*. All enzymatic tests were performed in triplicates, following the protocols described by Ciafardini and Zullo [21] with minor modifications.

The β-glucosidase activity was evaluated using two different substrates. The master plates of yeasts belonging to the four species were prepared using the MYGP agar medium supplemented with 0.1% (*w v^−1^*) esculin (Sigma-Aldrich, Milan, Italy) and 0.03% (*w v^−1^*) FeCl_3_ (Carlo Erba, Milan, Italy). After 48 h incubation at 30 °C, β-glucosidase activity was monitored visually based on the presence or absence of a dark halo around the colony, which was compared to that of the non-inoculated control plate. Each yeast was assigned a code based on the color of the halo, which indicates the enzymatic activity level as follows: N (no activity), light gray: L (low activity), black: H (high activity). The β-glucosidase activity of the same yeast cultures was confirmed in a 96-well microplate using the synthetic substrate, *p*-nitrophenylglucopyranoside (*p*-NPG) (Sigma-Aldrich, Milan, Italy). The microbial culture (100 µL; O.D._600_ = 0.70) in each well of the microplate (Falcon-Fisher Scientific, Milan, Italy) was incubated with 150 μL of 0.1 M phosphate buffer (pH 7) supplemented with 0.4% (*w v^−1^*) *p*-NPG at 30 °C for 180 min. The control included all reagents, except *p*-NPG. The absorbance of the reaction mixture was measured at 410 nm using a microplate reader (Fisher Scientific, Milan, Italy). The yeasts that exhibited enzymatic activity on both substrates were recorded as β-glucosidase producers.

The esterase activity was evaluated in a 96-well microplate using the 4-nitrophenyl acetate (4-NPA) substrate (Sigma-Aldrich). The yeast culture (70 μL; O.D._600_ = 0.8) in each well of the microplate was incubated with 70 μL of 0.5% (*w v^−1^*) 4-NPA prepared in methanol, and 70 μL of 0.1 M phosphate buffer (pH 7) at 30 °C for 180 min. The positive control was prepared by replacing the microbial cultures with 70 μL of porcine esterase (Sigma-Aldrich; 50 U mL^−1^ of phosphate buffer). The negative control lacked both yeast and esterase. The absorbance of the reaction mixture was measured at 410 nm using a microplate reader. The esterase analysis was also performed using the MYGP agar medium supplemented with NaCl (5 g L^−1^), CaCl_2_ (0.1 g L^−1^), and Tween 20 (5 mL L^−1^). The MYGP agar medium enriched with NaCl and CaCl_2_ was sterilized at 121 °C for 20 min and allowed to cool to 55 °C. Next, sterilized Tween 20 (Sigma-Aldrich) was added and mixed before the medium was poured into the plates. The plates inoculated with yeast strains were incubated at 30 °C for 10 days. The cultures were monitored daily for the presence of a cloudy halo around the colonies. The yeasts that exhibited enzyme activity in both tests were recorded as esterase producers.

The lipase activity was performed as described by Ciafardini et al. [22]. Briefly, 5 mL of the overnight stock culture of each yeast strain (O.D._600_ adjusted to ca. 0.8) was subjected to centrifugation at 9000× *g* for 5 min. Next, the culture pellet was suspended in 2 mL of 0.1 M phosphate buffer (pH 6) and incubated with 6 mL of filter-sterilized (Minisart NML-Sartorius, Göttingen, Germany) virgin olive oil. The negative control included all the components, except the yeast. Three repetitions were performed for each yeast strain. The samples were incubated at 30 °C for 7 days. The samples were vortexed daily for 1 min. The lipolytic activity was assessed through the titrimetric method for the determination of the olive oil free fatty acid content according to the European Community Regulation 1348/2013 [23].

The phenoloxidase activity was assessed using 7.5 mL of the overnight stock culture of each yeast culture (O.D._600_ adjusted to ca. 0.8). The overnight culture was subjected to centrifugation at 9000× *g* for 5 min. The culture pellet was suspended in 2 mL of 0.1 M phosphate buffer (pH 7) containing 100 mM of pyrocatechol (Sigma-Aldrich). The control included all the components, except yeast culture. The mixture was vortexed for 1 min and incubated at 30 °C for 60 min. The dark color intensity of the test group was visually compared with that of the control group.

To evaluate the peroxidase activity, 7.5 mL of the overnight stock culture (O.D._600_ adjusted to ca. 0.8) was pelleted. Next, the pelleted cells were incubated with 5 mL of a reaction mixture containing 0.30 mL of 4 % (*w v^−^^1^*) pyrogallol (Sigma-Aldrich), 0.30 mL of 1 % (*v v^−^^1^*) H_2_O_2_, and 4.4 mL of 0.1 M phosphate buffer (pH 7). The mixture was vortexed for 1 min and incubated at 30 °C for 60 min. The dark color intensity of the test group was compared with that of the control group.

The catalase activity was evaluated in a 96-well plate using yeast culture (150 µL) grown in MYGP broth overnight at 30 °C. The enzymatic activity was evaluated by adding 50 µL of 3% (*v v^−1^*) H_2_O_2_ in each well. The bubble production during 20 min incubation at 30 °C was visually evaluated and recorded as low or high catalase activity.

### 2.8. Analytical Indices

The free fatty acid concentration, peroxide values, and UV spectrophotometric indices (K_232_, K_270_, ΔK extinction coefficient K_266_, and K_274_) of the monovarietal and blended EVOO samples were evaluated at the beginning of the experimentation (zero time) and after 3 and 6 months of storage to assess their merceological class. All parameters were measured in triplicates for each sample according to European Community Regulation 1348/2013 [23].

### 2.9. Sensory Analysis

Sensory analysis was performed on EVOOs at the beginning of the experimentation (time zero) and after 3 and 6 months of storage by a fully trained analytical taste panel, which was recognized by the International Olive Oil Council (IOC). The panel test was established using an IOC standard profile sheet method [24]. Each panel member analyzed all samples during three different sessions. Three olive oil samples from each group were analyzed simultaneously by each panelist during three different sessions. The sample sets were randomly distributed among 10 assessors. The median values of the sensory data were calculated and the test supervisor chose a significance level of 5%.

### 2.10. Statistical Analyses

A priori one-way analysis of variance, followed by Tukey’s HSD (honest significant difference) test was performed using the Statgraphics computer program (Statgraphics, version 6, Manugistics, Inc., Rockville, MA, USA). The difference was considered statistically significant when the *p*-value was less than 0.01.

## 3. Results and Discussion

### 3.1. Microbiological and Physicochemical Characteristics of the Freshly Produced EVOOs

The microbiological analysis of the four freshly produced monovarietal EVOOs revealed a high abundance of yeasts and bacteria and a low abundance of mold in the EVOOs derived from the Coratina and Ravece varieties. The number of yeasts varied from 3.70 log CFU mL^−^^1^ (Leccino EVOO) to 5.41 log CFU mL^−^^1^ (Coratina EVOO), whereas that of bacteria varied from 2.28 log CFU mL^−1^ (Ravece EVOO) to 4.65 log CFU mL^−^^1^ (Coratina EVOO) (Table 1). The suspended solid content was high in the EVOOs extracted from the Coratina and Peranzana varieties. The water content in the four EVOOs varied from 0.22% (*w w^−^^1^*) (Leccino EVOO) to 0.45% (*w w^−^^1^*) (Coratina EVOO) (Table 1). This indicated that the high abundance of yeasts and bacteria in the Coratina EVOO was due to high suspended solid and water contents, which support the growth of microorganisms.

### 3.2. Physicochemical Analysis of the Monovarietal EVOOs used for Blending

The physicochemical analysis revealed that the suspended solid and water contents of the EVOOs analyzed were similar to those of the unfiltered veiled oils. The suspended solid content of freshly produced monovarietal EVOOs (Table 1) ranged from 0.070% (*w w^−^^1^*) to 0.092% (*w w^−^^1^*). However, the suspended solid content decreased by about 50% after the samples were subjected to sedimentation for 30 days (Table 2). The water content of the freshly produced EVOOs (Table 1) decreased when they were subjected to sedimentation for 30 days (Table 2). The physicochemical analysis performed at the beginning of the blending experiment (zero time) indicated that EVOO extracted from Leccino variety had the lowest water content (0.15%; *w w^−^^1^*), while EVOO extracted from Coratina variety had the highest water content (0.36%; *w w^−^^1^*) (Table 2). Ciafardini and Zullo [9] reported that low water content, which was observed in the EVOO derived from Leccino variety, prevents the deterioration of EVOO quality. Other studies have reported that high water content can adversely affect the shelf life of the product [12].

The chemical analysis of the monovarietal EVOOs performed at the initial phase of the blending indicated that the phenolic profiles vary depending on the olive variety from which the EVOOs were extracted. The EVOOs were grouped based on the total phenolic content as follows: low (Leccino EVOO with 179 mg tyrosol kg^−^^1^ oil), medium (Peranzana and Ravece EVOOs with 221 and 247 mg tyrosol kg^−^^1^ oil, respectively), and high (Coratina EVOO with 329 mg tyrosol Kg^−^^1^ oil) (Table 2). This indicated that some Italian olive varieties, including Coratina variety, normally produces EVOO with bitter-pungent taste and a high content of polar phenols, which are used to increase the phenolic content of other EVOOs through blending. When monovarietal EVOOs containing low phenolic content (Leccino EVOO) are blended with those containing high phenolic content (Coratina EVOO), the shelf life of the product increases because enhanced phenolic content increases the antioxidant activity. The consumer acceptance of EVOOs can be enhanced by blending EVOOs containing high phenolic content with a strong bitter and spicy taste (Coratina EVOO) and EVOOs with low phenolic content as the blended oil acquires a more balanced flavor. The monovarietal EVOOs used for blending are listed in Table 2. The monovarietal EVOOs extracted from Leccino and Peranzana varieties with a medium-low phenol content were blended with those extracted from Ravece and Coratina varieties with medium-high phenolic content and stored for a period of six months. The microbial dynamics and the quality index stability of EVOOs were assessed by analyzing the samples collected at the beginning of the experimentation (zero time) and after 3 and 6 months of storage.

### 3.3. Microbiological Analysis

The microbiological analysis of the four freshly produced monovarietal EVOOs revealed a high abundance of yeasts and bacteria and a low abundance of mold in the EVOOs extracted from the Coratina and Peranzana varieties (Table 1). The microbiological analysis of EVOO samples collected at the initial phase of blending (zero time) revealed a marked reduction in the abundance of bacteria and yeasts in all monovarietal EVOOs and a complete lack of mold (Table 3). The laboratory blending was performed using monovarietal EVOOs, which were subjected to sedimentation for 30 days. The reduction in the abundance of microbes can be mainly attributed to the sedimentation of micro-drops of vegetation water and solid particles, which are rich in microorganisms, toward the bottom of canisters. This was consistent with the results of previous studies [6]. However, the microbiota composition of the blended EVOOs was markedly different from that of the monovarietal EVOOs during storage. The number of bacteria markedly decreased during the first three months of storage. The bacteria were not detected in all EVOOs, except monovarietal EVOOs extracted from Peranzana and Coratina varieties. The reduction in yeast population during the first three months of EVOO storage was lesser than that in bacteria population in all samples, except the blended EVOOs extracted from Coratina and Ravece varieties. However, the microbiological analysis of EVOOs stored for six months revealed that the yeasts survived in all monovarietal EVOO samples, but not in the blended EVOOs (Table 3). The survival of yeasts in the blended EVOOs is lower than that in the monovarietal EVOOs, which is partly attributed to the unpredictable environments of blended oils that have depleted chemical content and other conditions that limit microbial activity. In a previously study has been demonstrated the survival of yeasts both in monovarietal oils and in mixture coming from the milling of different blends of olives produced by the same varieties [2]. The comparative analysis of the results reported in Table 3 and Table 1 and Table 2 suggested that the microbiota characteristics of oil freshly extracted from the fruits are determined at the beginning of the storage period. This is because of the presence of vegetation water and nutrients, which are favourable for microbial growth, during this period. In the case of blended EVOOs subjected to sedimentation, the growth of microbiota from each monovarietal EVOO is limited as they are exposed to harsh conditions, such as low water content and nutrient depletion.

### 3.4. Dynamics of Yeast Species Population during EVOOs Storage

The ribosomal (26S) D1/D2 region sequencing analysis of the most representative yeasts isolated from the EVOOs during storage allowed the identification of the following four yeast species: *Barnettozyma californica*, *Candida adriatica*, *Candida diddensiae*, and *Yamadazyma terventina*. These yeast species were identified in all EVOOs samples analyzed at the beginning of the experiment (zero time). During storage, the yeast species were identified in the monovarietal EVOOs but not in the blended EVOOs for lack of colonies to be examined (Table 4). The maximum number of yeast species identified in the EVOO samples was as follows: two in EVOOs extracted from Leccino and Ravece varieties, three in EVOO extracted from Peranzana, and four in EVOO extracted from Coratina. The prevalence of yeast species in the EVOOs varied from 60% to 98% depending on the olive variety. *B. californica* and *C. adriatica* were the abundant yeast species in the monovarietal EVOOs derived from Leccino and Ravece during the entire storage period, respectively. Contrastingly, *B. californica* and *C. diddensiae* were the abundant yeast species in the EVOOs derived from Peranzana and Coratina during the first months of storage, respectively, whereas *Y. terventina* was abundant in both EVOOs at the end of the storage period. The results reported in Table 4 are consistent with those of our previous study, which demonstrated the predominance of single yeast species in monovarietal oils [17].

### 3.5. Enzymatic Activity of the Yeast Species

The activities of β-glucosidase, esterase, lipase, peroxidase, phenoloxidase, and catalase were evaluated in the yeast isolates identified at the species level (Table 5). These enzymes are reported to be involved in the reduction of phenols and the production of new compounds, which determine the oil quality [25,26].

The proportion of β-glucosidase-producing yeast strains varied according to the species as follows: *B. californica* (34%), *C. diddensiae* (90%), *C. adriatica*, and *Y. terventina* (100%). Additionally, the proportion of strains producing β-glucosidase was highest among *Y. terventina* strains (Table 5). β-glucosidase is an important enzyme in olive oil as it hydrolyzes oleuropein, a bitter-tasting glucoside, into aglycone and glucose, which improve the sensory profile of bitter oils [10].

The production of esterase was observed in all the *C. diddensiae* strains among which 70% exhibited strong enzymatic activity.

The lipase activity was observed in 90%, 80%, 67%, and 12% of *Y. terventina*, *C. diddensiae*, *B. californica*, and *C. adriatica* strains, respectively. Strong lipase activity was exhibited by 40% *C. diddensiae* and 20% *Y. terventina* strains. Lipase (triacylglycerol acylhydrolase) and esterase (carboxylic-ester hydrolase) hydrolyze hydrophobic long- and short-chain carboxylic acid esters, respectively. Lipase catalyzes the hydrolysis of ester bonds at the interface between an insoluble substrate phase (olive oil) and the aqueous phase, whereas esterase catalyzes the hydrolysis of ester bonds of water-soluble substrates. Esterase is involved in the debittering process of the oil through the hydrolysis of aglycones derived from the hydrolysis of oleuropein, which partly preserves the bitter taste of the starting compound. Lipase hydrolyzes the EVOO triglycerides and enhances the free fatty acid (FFA) level [27,28]. The lipase in some yeast strains isolated from the EVOOs promotes lipolytic activity during storage [29]. One of the major factors that affect olive oil quality is acidity, which affects EVOO stability [30]. The European Community Regulation 1348/2013 [23] recommends that EVOO must contain less than 0.80% of total FFAs (expressed as oleic acid).

The oxidase activity mediated by peroxidase and phenoloxidase was most evident in the *C. diddensiae*, *Y. terventina,* and *B. californica* strains. However, the proportion of *C. adriatica* exhibiting peroxidase and phenoloxidase activities was low. In contrast to other enzymes, catalase activity was observed in all the yeast strains (Table 5). The oxidase enzymes, including peroxidases and polyphenol oxidases, oxidize phenolic compounds and polyphenols and affect the sensorial quality of EVOOs during storage [10].

### 3.6. Quality Evolution during EVOOs Storage

The quality of the EVOOs during storage was assessed by comparing the results acquired from the analytical indices and the sensorial tests with the limit values established by the European Community Regulation 1348/2013 for the EVOO class [23]. All the samples of monovarietal oils used in the blending tests were initially EVOO, although some analytical indices at the beginning of experimentation (zero time) were high partly due to the presence of *Bactrocera oleae* (Rossi) [28]. This was useful to investigate the time course of quality index stability and the ability of EVOOs to maintain the high initial analytical parameters that enables the maintenance of merceological class EVOO, during storage. The chemical analysis associated with quality indices indicated that the quality of all monovarietal EVOOs, except Ravece EVOO, deteriorated and did not satisfy the recommended merceological parameters of EVOO after the third month of storage. In contrast, the analytical indices of the blended EVOOs indicated stability and thus could be classified as EVOO after six months of storage (Table 6).

The sensorial analysis results of monovarietal EVOOs were consistent with the analytical index results, which indicated that the EVOOs extracted from Leccino, Peranzana, and Coratina varieties exhibited muddy sediment defect (Table 7). The comparative analysis of these results and the results of previous studies suggested that blended EVOOs exhibit better stability than the monovarietal EVOOs. This can be attributed to the microbiota composition, and to the total polar phenolic compound and water contents.

The comparative analysis of quality index results (reported in Table 6 and Table 7) and other results indicated that the low stability exhibited by monovarietal EVOO extracted from Leccino may be due to its low phenolic content (Table 2) and due to the high esterase and oxidase activities in predominant yeasts, including *C. diddensiae* strains (Table 4 and Table 5) [7,8]. The quality deterioration of the monovarietal EVOOs extracted from Peranzana and Coratina may be due to the enzymatic activities in the *C. diddensiae* and *Y. terventina* yeast strains and partly due to the presence of bacteria (Table 3) [31]. The data reported in Table 2 and Table 6 indicated that the high phenolic content of EVOOs extracted from Coratina could not inhibit the enzymatic activity, which increased the levels of FFAs during storage. This may be due to the high water content, which promotes the activity of lipase derived from microorganisms or fruits. The lipase activity of some oil-borne yeast is reported to be maximum when more than 0.25% of water is present in the olive oil [29]. Among the monovarietal EVOOs, only Ravece EVOO was stable during storage. This may be due to the medium-high content of polar phenolic compounds and low water content in the Ravece EVOO (Table 2). Additionally, the low abundance of yeasts (majorly *C. adriatica*) may also have contributed to the stability of Ravece EVOO during storage as the enzymatic activity in this species does not adversely affect oil quality (Table 3, Table 4 and Table 5).

## 4. Conclusions

Blending is a widespread practice in EVOO manufacturing companies. It is used for the production of sufficient quantities of EVOO with a balanced taste to meet the demands of the global market. In this study, we demonstrated that the microbiota, which was established in the EVOOs immediately after extraction, survive well in the monovarietal EVOOs but not in the blended EVOOs accomplished after one month of storage. Several oil-borne yeasts derived from healthy olives are desirable as they improve some sensory characteristics of the oil. However, some yeasts are often derived from damaged olives and can impair the oil quality under favourable conditions, such as high-water content and low phenolic concentration. These observations indicate that the blended EVOOs are more stable than the monovarietal EVOOs due to the limited number of microorganisms and their low metabolic activity during storage.

## Figures and Tables

**Table 1:** Microbial and physical characteristics of newly extracted monovarietal extra virgin olive oils (EVOOs.).

Olive Variety	Microbial Content (Log CFU mL^−^^1^)		Physical Characteristics (%, *w w*^−1^)		
	Yeast	Bacteria	Molds	Suspended Solid	Water content
Leccino	3.70 ± 0.29 ^c^	2.64 ± 0.13 ^c^	0	0.075 ± 0.003 ^b^	0.22 ± 0.02 ^c^
Peranzana	4.73 ± 0.30 ^b^	3.62 ± 0.15 ^b^	0	0.083 ± 0.005 ^ab^	0.30 ± 0.01 ^b^
Coratina	5.41 ± 0.60 ^a^	4.65 ± 0.09 ^a^	0.76 ± 0.07	0.092 ± 0.008 ^a^	0.45 ± 0.04 ^a^
Ravece	4.31 ± 0.19 ^b^	2.28 ± 0.20 ^c^	1.46 ± 0.34	0.070 ± 0.004 ^b^	0.34 ± 0.02 ^b^

The values reported in the column with different letters are significantly different from one another at *p* < 0.01.

**Table 2:** Physico-chemical characteristics of the monovarietal extra virgin olive oils (EVOOs) used in the blending trials.

Parameters	Leccino	Peranzana	Coratina	Ravece
Solid content (%, *w w^−1^*)	0.035 ± 0.003	0.038 ± 0.005	0.040 ± 0.008	0.035 ± 0.004
Water content (%, *w w*^−1^)	0.15 ± 0.02 ^c^	0.25 ± 0.01 ^b^	0.36 ± 0.04 ^a^	0.24 ± 0.02 ^b^
Polar phenols (mg Kg^−1^)				
OH-Tyr	8 ± 0.12 ^b^	7 ± 0.15 ^b^	18 ± 0.11 ^a^	6 ± 0.07 ^b^
Tyr	12 ± 0.19 ^a^	7 ± 0.11 ^b^	12 ± 0.11 ^a^	5 ± 0.09 ^b^
3,4-DHPEA-EDA	22 ± 0.22 ^c^	50 ± 0.31 ^ab^	65 ± 0.38 ^a^	47 ± 0.19 ^b^
p-HPEA-EDA	39 ± 0.39 ^b^	47 ± 0.41 ^b^	85 ± 0.30 ^a^	45 ± 0.19 ^b^
Lignan	36 ± 0.29 ^ab^	25 ± 0.11 ^b^	41 ± 0.11 ^a^	46 ± 0.19 ^a^
O-Agl	10 ± 0.12 ^b^	11 ± 0.15 ^b^	18 ± 0.11 ^a^	17 ± 0.19 ^a^
L-Agl	5 ± 0.10 ^ab^	6 ± 0.11 ^a^	5 ± 0.2 ^ab^	3 ± 0.09 ^b^
Total phenols ^1^	179 ± 26 ^c^	221 ± 32 ^bc^	329 ± 48 ^a^	247 ± 36 ^b^

OH-Tyr, Hydroxytyrosol; Tyr, Tyrosol; DMO-da, Dialdeid form of decarboxymethyl elenolic acid linked to hydroxytyrosol; DML-da, Dialdeid form of decarboxymethyl elenolic acid linked to tyrosol; O-Agl, Oleuropein aglycone; L-Agl, Ligstroside aglycone; ^1^; concentration of total phenols expressed as mg tyrosol per kg EVOO. The values in line with different letters are significantly different from one another at *p* < 0.01.

**Table 3:** Microbiological analysis of monovarietal and blended extra virgin olive oils (EVOOs) during storage.

Olive Variety	Zero Time			Three Months		Six Months	
	Yeast	Bacteria	Molds	Yeast	Bacteria	Yeast	Bacteria
Leccino (L)	2.38 ± 0.20 ^a1^	1.82 ± 0.03	0	1.94 ± 0.18 ^b^	0	1.72 ± 0.23 ^b^	0
Peranzana (P)	2.73 ± 0.25 ^a^	2.80 ± 0.06 ^a^	0	1.50 ± 0.10 ^ab^	1.95 ± 0.03 ^b^	1.19 ± 0.10 ^b^	0
Coratina (C)	2.19 ± 0.40 ^a^	2.11 ± 0.09 ^a^	0	1.82 ± 0.02 ^b^	0.75 ± 0.01 ^b^	1.32 ± 0.15 ^c^	0
Ravece (R)	1.76 ± 0.34 ^a^	1.08 ± 0.05	0	0.90 ± 0.12 ^b^	0	0.85 ± 0.10 ^b^	0
LP (ratio 1:1)	2.01 ± 0.17 ^a^	0.70 ± 0.13	0	0.50 ± 0.01 ^b^	0	0	0
CR (ratio 1:1)	1.46 ± 0.09	1.4 ± 0.23	0	0	0	0	0
LPR (ratio 1:1:1)	1.89 ± 0.25 ^a^	1.80 ± 0.32	0	0.40 ± 0.01 ^b^	0	0	0
LPCR (ratio 1:1:1:1)	1.99 ± 0.05 ^a^	1.08 ± 0.13	0	0.78 ± 0.02 ^b^	0	0	0

^1^, Mean ± standard deviation Log CFU mL^−^^1^; LP, Blend Leccino + Peranzana; CR, Blend Coratina + Ravece; LPR, Blend Leccino + Peranzana + Ravece; LPCR, Blend Leccino + Peranzana + Coratina + Ravece. The values of yeasts or bacteria, respectively, reported in line with different letters are significantly different from one another at *p* < 0.01.

**Table 4:** Dynamic of yeast species populations in monovarietal and blended extra virgin olive oils (EVOOs) during storage.

Olive Variety	Chromogenic Yeast Group	Zero Time		Three Months		Six Months	
		Yeast Species	Prevalence (%)	Yeast Species	Prevalence (%)	Yeast Species	Prevalence (%)
Leccino (L)	4–5	*B. californica*	98	*B. californica*	40	*B. californica*	4
	3	*C. diddensiae*	2	*C. diddensiae*	60	*C. diddensiae*	96
Peranzana (P)	4–5	*B. californica*	90	*B. californica*	28	*B. californica*	7
	1–2	*C. adriatica*	5	*C. adriatica*	12	*C. adriatica*	14
	6	*Y. terventina*	5	*Y. terventina*	60	*Y. terventina*	79
Coratina (C)	3	*C. diddensiae*	83	*C. diddensiae*	55	*C. diddensiae*	5
	1–2	*C. adriatica*	9	*C. adriatica*	15	*C. adriatica*	9
	6	*Y. terventina*	5	*Y. terventina*	30	*Y. terventina*	86
	4–5	*B. californica*	3	*B. californica*	0	*B. californica*	0
Ravece (R)	1–2	*C. adriatica*	60	*C. adriatica*	64	*C. adriatica*	75
	6	*Y. terventina*	40	*Y. terventina*	36	*Y. terventina*	25
LP (ratio 1:1)		*B.c., C.a., Y.t.*	50, 45, 5	nd		nd	
CR (ratio 1:1)		*C.d., C.a., B.c., Y.t*	35, 32, 20, 13	nd		nd	
LPR (ratio 1:1:1)		*C.a., B.c., Y.t.*	40, 30, 30	nd		nd	
LPCR (ratio 1:1:1:1)		*B.c., C.a., Y.t., C.d.*	30, 32, 23, 15	nd		nd	

*B.c., Barnettozyma californica; C.a., Candida adriatica*; *C.d., Candida diddensiae; Y.t., Yamadazyma terventina*. Nd, Not detected. LP, Blend Leccino + Peranzana; CR, Blend Coratina + Ravece; LPR, Blend Leccino + Peranzana + Ravece; LPCR, Blend Leccino + Peranzana + Coratina + Ravece.

**Table 5:** Enzymatic activity in yeast species predominant in the extra virgin olive oils (EVOOs).

Yeast Species	No. Isolates		β-Glucosidase		Esterase			Lipase			Peroxidase			Phenoloxidase			Catalase		
		N	L	H	N	L	H	N	L	H	N	L	H	N	L	H	N	L	H
*B. californica*	9	66 ^1^	34	0	22	33	45	33	60	7	0	0	100	0	100	0	0	66	34
*C. adriatica*	16	0	87	13	63	25	12	88	12	0	12	75	13	50	50	0	0	75	25
*C. diddensiae*	20	10	80	10	0	30	70	20	40	40	0	80	20	0	70	30	0	60	40
*Y. terventina*	15	0	80	20	13	60	27	20	60	20	0	80	20	0	60	40	0	60	40

^1^, %. N, no activity; L, low activity, H, high activity. *B. californica*, *Barnettozyma californica*; *C. adriatica*, *Candida adriatica*; *C. diddensiae*, *Candida diddensiae*; *Y. terventina*, *Yamadazyma terventina*.

**Table 6:** Analytical indices of monovarietal and blended extra virgin olive oils (EVOOs) evaluated during six months of storage.

Olive Cultivar	Free Fatty Acid (% oleic acid)			Peroxide Value (meq O_2_ Kg^−1^)			K_232_			K_270_			ΔK			EVOO Merceological Class		
Leccino (L)	0 ^1^	3	6	0	3	6	0	3	6	0	3	6	0	3	6	0	3	6
	0.70 ^2^	0.70	0.70	8.77 ^c^	11.30 ^b^	13.80 ^a^	1.956	2.111	**2.592** ^3^	0.129	0.124	0.100	−0.001	−0.001	−0.001	+	+	-
	(0.06)	(0.07)	(0.09)	(0.11)	(0.14)	(0.21)	(0.002)	(0.010)	(0.005)	(0.001)	(0.004)	(0.001)						
Peranzana (P)	0.75	0.74	0.74	7.93 ^c^	10.00 ^b^	15.80 ^a^	2.018 ^b^	2.281 ^ab^	**2.772** ^a^	0.176	0.190	0.166	−0.001	0.000	−0.001	+	+	-
	(0.01)	(0.01)	(0.02)	(0.40)	(0.50)	(0.39)	(0.003)	(0.005)	(0.010)	(0.004)	(0.006)	(0.006)						
Coratina (C)	0.70 ^c^	0.80 ^b^	**0.86** ^a^	6.97 ^c^	9.20 ^b^	12.90 ^a^	1.817 ^b^	1.989 ^ab^	2.152 ^a^	0.160	0.141	0.122	−0.001	−0.001	−0.001	+	+	-
	(0.06)	(0.01)	(0.01)	(0.25)	(0.20)	(0.35)	(0.003)	(0.005)	(0.007)	(0.003)	(0.004)	(0.010)						
Ravece (R)	0.51	0.50	0.53	9.90 ^c^	10.80 ^b^	14.60 ^a^	1.807	2.075	2.278	0.145	0.141	0.140	−0.001	−0.001	−0.001	+	+	+
	(0.01)	(0.01)	(0.01)	(0.10)	(0.10)	(0.11)	(0.002)	(0.002)	(0.003)	(0.002)	(0.002)	(0.007)						
LP (ratio 1: 1)	0.79	0.78	0.78	8.00 ^c^	9.00 ^b^	13.90 ^a^	1.934 ^b^	2.066 ^ab^	2.494 ^a^	0.158	0.153	0.150	−0.001	−0.001	0.000	+	+	+
	(0.01)	(0.02)	(0.02)	(0.20)	(0.21)	(0.22)	(0.002)	(0.005)	(0.012)	(0.001)	(0.002)	(0.008)						
CR (ratio 1:1)	0.68	0.68	0.70	7.03 ^b^	7.80 ^b^	11.90 ^a^	1.847 ^b^	2.015 ^ab^	2.312 ^a^	0.168	0.159	0.184	−0.002	−0.001	−0.002	+	+	+
	(0.02)	(0.03)	(0.03)	(0.15)	(0.14)	(0.17)	(0.001)	(0.002)	(0.005)	(0.002)	(0.001)	(0.002)						
LPR (ratio 1:1:1)	0.67	0.67	0.71	9.13 ^b^	8.90 ^b^	13.50 ^a^	1.917 ^b^	2.008 ^b^	2.483 ^a^	0.120	0.154	0.136	−0.001	−0.002	0.000	+	+	+
	(0.01)	(0.03)	(0.01)	(0.21)	(0.11)	(0.31)	(0.003)	(0.004)	(0.009)	(0.001)	(0.002)	(0.008)						
LPCR (ratio 1:1:1:1)	0.62	0.63	0.62	8.97 ^b^	8.90 ^b^	12.20 ^a^	1.910 ^b^	1.954 ^b^	2.350 ^a^	0.164	0.151	0.137	−0.008	−0.002	−0.001	+	+	+
	(0.02)	(0.07)	(0.03)	(0.25)	(0.24)	(0.27)	(0.002)	(0.008)	(0.010)	(0.002)	(0.003)	(0.001)						
Limit for the EVOO merceological class	≤0.80			≤20			≤2.50			≤0.22			≤0.010					

^1^, Months of storage. ^2^, Mean ± standard deviation (in brackets). ^3^, Bold values are higher than the limit values established by the European Community Regulation 1348/2013 for the EVOO class. +, EVOO merceological class; -, Excluded from the commercial class EVOO. LP, Blend Leccino + Peranzana; CR, Blend Coratina + Ravece; LPR, Blend Leccino + Peranzana + Ravece; LPCR, Blend Leccino + Peranzana + Coratina + Ravece. The values of each parameter in line with different letters are significantly different from one another at *p* < 0.01.

**Table 7:** Sensory attributes of monovarietal and blended extra virgin olive oils (EVOOs) during storage.

Olive Cultivar	Fruitiness			Bitterness			Pungency			Defect			Merceologicalclass		
	0 ^1^	3	6	0	3	6	0	3	6	0	3	6	0	3	6
Leccino (L)	2.8 ^a,2^	2.0 ^b^	2.0 ^b^	1.3	1.2	1.0	1.7 ^a^	1.5 ^ab^	1.0 ^b^	MS	MS	MS	EVOO	-	-
	(0.03)	(0.04)	(0.01)	(0.01)	(0.04)	(0.03)	(0.02)	(0.03)	(0.01)						
Peranzana (P)	3.3 ^a^	2.3 ^b^	2.0 ^b^	2.9 ^a^	2.5 ^b^	2.0 ^b^	3.7 ^a^	3.0 ^ab^	2.5 ^b^	0	0	MS	EVOO	EVOO	-
	(0.05)	(0.02)	(0.04)	(0.03)	(0.05)	(0.03)	(0.02)	(0.02)	(0.01)						
Coratina (C)	2.8 ^a^	2.1 ^b^	2.0 ^b^	4.3 ^a^	2.8 ^b^	1.5 ^c^	4.4 ^a^	3.0 ^b^	2.0 ^c^	0	MS	MS	EVOO	-	-
	(0.02)	(0.01)	(0.02)	(0.03)	(0.04)	(0.03)	(0.01)	(0.01)	(0.03)						
Ravece (R)	4.4	4.3	3.8	4.1	3.8	3.5	4.8 ^a^	4.0 ^ab^	3.5 ^b^	0	0	0	EVOO	EVOO	EVOO
	(0.05)	(0.03)	(0.03)	(0.04)	(0.04)	(0.01)	(0.02)	(0.03)	(0.03)						
L P (ratio 1:1)	3.3	2.8	2.8	2.5 ^a^	2.0 ^ab^	1.8 ^b^	3.2 ^a^	3.0 ^a^	2.2 ^b^	0	0	0	EVOO	EVOO	EVOO
	(0.03)	(0.01)	(0.03)	(0.05)	(0.04)	(0.01)	(0.02)	(0.03)	(0.02)						
C R (ratio 1:1)	3.6	3.3	3.0	2.5 ^a^	2.0 ^b^	2.0 ^b^	3.0	3.0	3.0	0	0	0	EVOO	EVOO	EVOO
	(0.04)	(0.02)	(0.03)	(0.03)	(0.01)	(0.03)	(0.02)	(0.01)	(0.02)						
LPR (ratio 1:1:1)	3.5 ^a^	3.5 ^a^	3.0 ^b^	2.4	2.4	2.0	2.7 ^a^	2.0 ^b^	2.0 ^b^	0	0	0	EVOO	EVOO	EVOO
	(0.06)	(0.04)	(0.05)	(0.03)	(0.02)	(0.04)	(0.02)	(0.02)	(0.03)						
LPCR (ratio 1:1:1:1)	4.5 ^a^	4.0 ^b^	4.0 ^b^	3.9	3.5	3.5	4.4 ^a^	3.8 ^b^	4.0 ^ab^	0	0	0	EVOO	EVOO	EVOO
	(0.06)	(0.04)	(0.03)	(0.03)	(0.04)	(0.01)	(0.02)	(0.01)	(0.04)						

^1^ Months of storage. ^2^ Median of the values ± standard deviation (in brackets). LP, Blend Leccino + Peranzana; CR, Blend Coratina + Ravece; LPR, Blend Leccino + Peranzana + Ravece; LPCR, Blend Leccino + Peranzana + Coratina + Ravece. MS, Muddy Sediment defect. The values of each parameter in line with different letters are significantly different from one another at *p* < 0.05.
